# Association between advanced lung cancer inflammation index and osteoporosis in patients with type 2 diabetes mellitus: evidence from NHANES

**DOI:** 10.3389/fendo.2024.1421696

**Published:** 2024-11-25

**Authors:** Yifeng Xu, Zhaoqi Yan, Liangji Liu

**Affiliations:** ^1^ School of Clinical Medicine, Jiangxi University of Chinese Medicine, Nanchang, Jiangxi, China; ^2^ Guang'anmen Hospital, China Academy of Chinese Medical Sciences, Beijing, China; ^3^ Affiliated Hospital of Jiangxi University of Chinese Medicine, Nanchang, Jiangxi, China

**Keywords:** advanced lung cancer inflammation index, type 2 diabetes mellitus, osteoporosis, cross-sectional study, NHANES

## Abstract

**Background:**

Previous studies have shown a significantly increased prevalence of osteoporosis (OP) in patients with type 2 diabetes mellitus (T2DM), which is closely associated with inflammation and nutrition. This study aimed to investigate the relationship between the advanced lung cancer inflammation index (ALI) and OP in patients with T2DM.

**Methods:**

This cross-sectional analysis was conducted based on data from middle-aged and older adults aged 50 years and older with T2DM from the National Health and Nutrition Examination Survey (NHANES).Weighted multivariable logistic regression and linear regression were utilized to investigate the correlation between the ALI and OP with femur bone mineral density (BMD) in individuals with T2DM. Restricted cubic splines (RCS) were employed to assess potential nonlinear relationships, and receiver operating characteristic (ROC) curves were used to evaluate diagnostic accuracy.

**Results:**

A total of 1596 patients with T2DM were included in this study, among whom 736 had OP. After adjusting for covariates, the multivariable logistic regression model showed that compared to participants in the fourth quartile of log2-transformed ALI, those in the first quartile had an increased prevalence of OP in T2DM (OR = 1.95, 95% CI=1.28-2.96, p < 0.01). The multivariable linear regression model indicated that a low log2-transformed ALI is associated with a low femur BMD.RCS demonstrated a linear dose-response relationship between the ALI index and OP in T2DM (p = 0.686), with the area under the ROC curve being 0.57 (95% CI: 0.54-0.60, p < 0.001), and the optimal cutoff value was 6.04.

**Conclusion:**

Our findings indicate that low levels of ALI are independently associated with an increased prevalence of OP in middle-aged and older adults with T2DM in the United States. ALI may serve as a potential biomarker for assessing the prevalence of OP in middle-aged and older adults with T2DM.

## Introduction

Osteoporosis (OP) is a metabolic skeletal condition characterized by diminished bone density and degradation of bone microstructure, ultimately resulting in heightened bone fragility and vulnerability to fractures (1). This condition substantially amplifies the likelihood of disability, hospitalization, and mortality (2, 3). In the United States, approximately 10.2 million individuals aged 50 and above are affected by OP, a number projected to swell to 13.46 million by 2030 (4), imposing significant economic burdens on both individuals and society.

Diabetes, as a disease posing a significant threat to global health, with over 90% of cases being type 2 diabetes mellitus (T2DM) (5), has recently shown a high prevalence of OP among T2DM patients, reaching up to 27.67% (6). Furthermore, OP is increasingly acknowledged as a consequence of diabetes (7). Presently, bone mineral density (BMD) ascertained through dual-energy X-ray absorptiometry (DXA) stands as the standard technology for clinically evaluating bone strength. However, there are indications that it might underestimate fracture risk in diabetic patients (8). Additionally, several studies indicate that fractures in diabetic patients have worse outcomes compared to normal controls (9, 10). Importantly, OP is often challenging to detect before fragility fractures occur, leading to delayed diagnosis and treatment (11). Therefore, identifying new and beneficial early screening tools for OP in T2DM patients is crucial.

Previous studies have indicated that the pathogenesis of OP involves various factors, including oxidative stress, inflammation, nutritional status, physical activity, and their potential interactions (12–14). Overproduction of reactive oxygen species (ROS) triggers abnormalities in osteoblasts (15), heightened levels of pro-inflammatory cytokines affect bone resorption (16), and increase in the number of osteoclasts (17). The advanced lung cancer inflammation index (ALI) is derived by multiplying body mass index (BMI) with serum albumin and dividing it by the neutrophil-to-lymphocyte ratio (NLR), reflecting nutritional and inflammatory status (18). It has been used not only in lung cancer patients but also has shown close associations with the prognosis of hypertension and heart failure in recent years (19, 20). Moreover, it is calculated from objective parameters obtained through laboratory tests, making it clinically practical. However, the role of ALI in OP among patients with T2DM remains unclear. Therefore, this study utilizes the National Health and Nutrition Examination Survey (NHANES) database to explore the correlation between ALI and OP in patients with T2DM.

## Materials and methods

### Data source and participants

The NHANES survey adopts a sophisticated, multi-stage probability sampling framework overseen by the Centers for Disease Control and Prevention (CDC). Its primary objective is to evaluate the health and nutritional condition of a nationally representative cross-section of adults and children in the United States. Presently, data collection and dissemination follow a biennial pattern, encompassing demographic, dietary, physical examination, laboratory, and questionnaire data. Approval for the NHANES protocol has been granted by the Research Ethics Review Board of the National Center for Health Statistics, and written informed consent has been secured from all participants.

**Figure 1:**
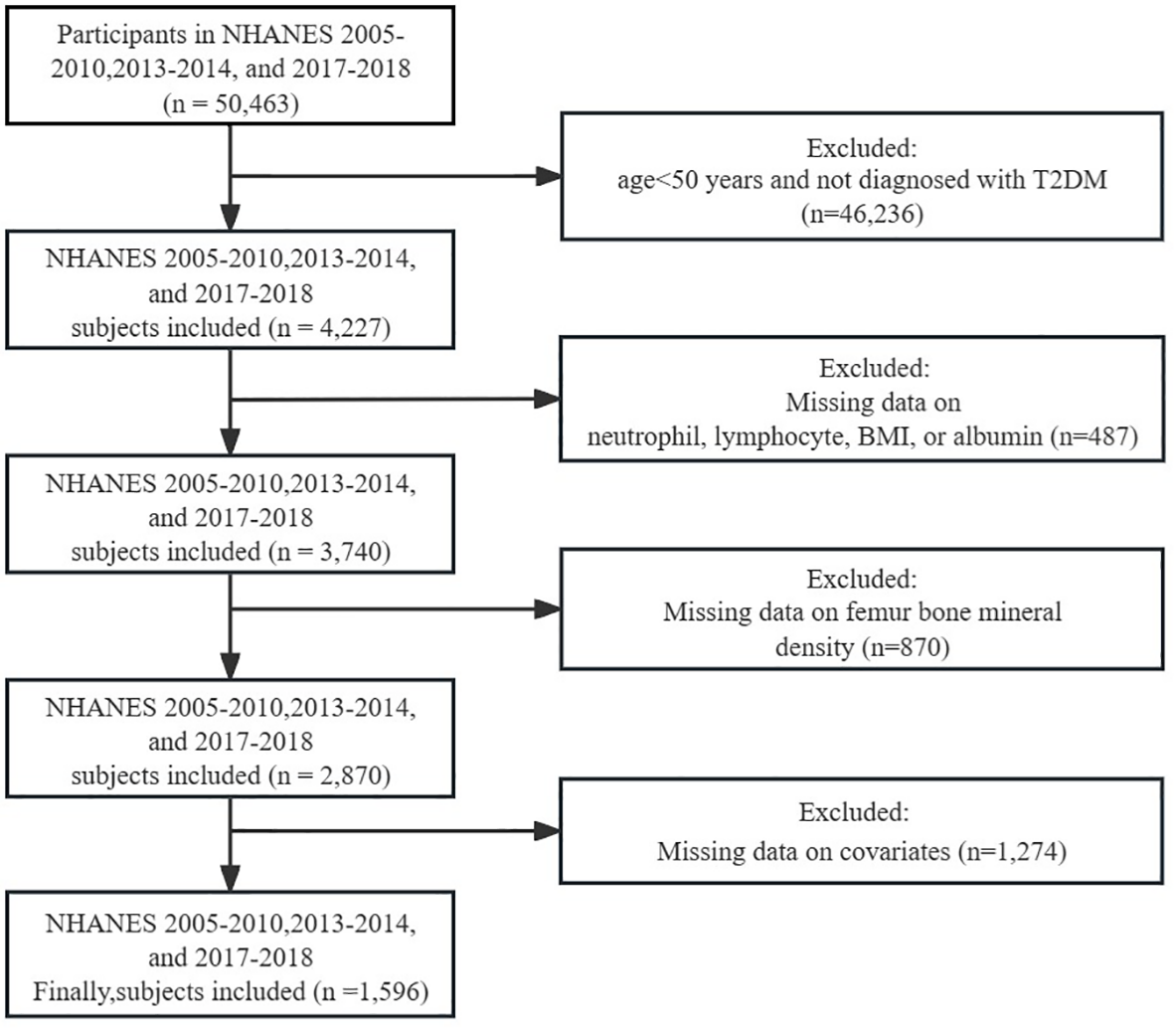
Flow chart of this study.

For this cross-sectional investigation, data from five NHANES cycles spanning from 2005-2010, 2013-2014, and 2017-2018 were extracted as the foundation for analysis. Participants were disqualified if they: (1) were under 50 years of age and not diagnosed with T2DM; (2) had missing data for neutrophils, lymphocytes, BMI, or albumin; (3) had missing data for femur BMD; (4) had missing data for covariates. Ultimately, a total of 1596 individuals satisfied the inclusion criteria for this investigation (**Figure 1**).

### Assessment of exposure

The calculation formula for ALI is as follows: ALI = BMI (kg/m^2^) × albumin level (g/dL)/NLR, where NLR is determined from the ratio of neutrophil count to absolute lymphocyte count. Due to the right-skewed distribution of ALI, log2 transformation was applied during the regression analysis (**Supplementary Figure S1**). According to quartiles, log2-ALI levels were divided into four groups,Q1(<5.406)、Q2(>5.406,≤5.911)、Q3(>5.911, ≤6.361)、Q4(>6.361).

### Assessment of outcome

We delineated T2DM in accordance with the standards established by the International Diabetes Federation (IDF): (1) medically confirmed diabetes; (2) utilization of oral hypoglycemic agents or insulin; (3) random/oral glucose tolerance test with a 2-hour blood glucose level ≥11.1 mmol/L; (4) fasting blood glucose ≥7.0 mmol/L; (5) glycated hemoglobin (HbA1c) ≥6.5%. The definition of OP is based on the classification criteria established by the World Health Organization, which defines it as a BMD value in any region of the femur that is 2.5 standard deviations (SD) or more below the mean value of a young adult reference population (21); DXA measurements of BMD were performed using a Hologic QDR-4500A fan-beam densitometer (Hologic, Inc., Bedford, Massachusetts). Total femur, femoral neck, trochanter, and intertrochanteric regions were included in the assessment of OP, and respective reference ranges were obtained from previously published NHANES III reports (22).

### Covariates

The factors considered in this study encompass age, sex, race, marital status, education attainment, smoking status, poverty income ratio (PIR), BMI, physical activity, aspartate aminotransferase (AST), alanine aminotransferase (ALT), alkaline phosphatase (ALP), serum creatinine, serum uric acid, blood urea nitrogen, serum calcium, serum phosphorus, glycated hemoglobin (HbA1c), triglycerides, total cholesterol, cardiovascular disease (CVD), hypertension, and cancer. Race is categorized into 4 groups: non-Hispanic White, non-Hispanic Black, Mexican American, or Other; marital status is divided into 3 categories: married/cohabiting, never married, and widowed/divorced/separated; education attainment is classified into 5 categories: less than 9th grade, 9-11th grade, high school graduate/GED certificate, some college or associate’s degree, and college graduate or higher. Smoking status is divided into 3 groups: never smokers, former smokers, and current smokers; never smokers are defined as smoking fewer than 100 cigarettes in their lifetime, former smokers are defined as smoking more than 100 cigarettes but currently not smoking, and current smokers are defined as smoking more than 100 cigarettes and currently smoking. PIR is categorized as ≤1.3, 1.3-3.5, and >3.5. Physical activity is converted into metabolic equivalent (MET) scores, where the weekly time spent on each leisure activity reported by participants is multiplied by the MET value of that activity to obtain weekly MET-minute index, with MET minutes/week ≥500 considered as physically active and MET minutes/week <500 considered as physically inactive, following the criteria recommended by NHANES (23). CVD is defined based on the presence of congestive heart failure (CHF)/coronary heart disease (CHD)/angina/heart attack/stroke. Hypertension is determined by self-reported hypertension, average systolic blood pressure ≥140 mmHg, average diastolic blood pressure ≥90 mmHg, or the use of antihypertensive medications. Cancer is defined based on whether a physician has ever told you that you have cancer or malignancy.

### Statistical analysis

Our study accounted for the intricate sampling design and sample weights utilized in NHANES. Categorical variables were depicted as numbers and percentages (%), whereas continuous variables were expressed as mean ± standard error (SE). Weighted t-tests were employed for continuous variables, and weighted chi-square tests were utilized to discern significant disparities in population characteristics among categorical variables.

We employed weighted univariate and multivariate logistic regression models to explore the relationship between ALI and OP in the T2DM population, with findings presented as odds ratios (ORs) and corresponding 95% confidence intervals (CIs). Weighted multivariate linear regression models were employed to assess the association between ALI and femur BMD in T2DM, with results presented as β coefficients and 95% CIs. Regression models were constructed by gradually adjusting covariates: the crude model was unadjusted, model 1 adjusted for age, sex, and race; model 2 further adjusted for smoking status, marital status, education level, PIR, and physical activity on the basis of model 1; model 3 additionally adjusted for AST, ALT, ALP, serum creatinine, serum uric acid, blood urea nitrogen, serum calcium, serum phosphorus, HbA1c, triglycerides, total cholesterol, CVD, hypertension, and cancer. Additionally, a linear trend test was conducted by converting the categorical variables of log2-ALI quartiles into continuous variables. To investigate the nonlinear correlation between ALI and OP, as well as femur BMD in T2DM, restricted cubic splines (RCS) with three knots (at the 10th, 50th, and 90th percentiles) were utilized to examine the dose-response relationship in Model 3. The area under the receiver operating characteristic (ROC) curve was employed to assess the predictive accuracy of ALI for identifying OP patients within the T2DM population and to ascertain the optimal cutoff point. In addition, subgroup and interaction analyses were conducted based on age (<65 years or ≥65 years), sex (male or female), smoking status (smoker or non-smoker), physical activity (active or inactive), hypertension (yes or no), and cardiovascular disease (CVD) (yes or no).

Finally, we conducted a series of sensitivity analyses, specifically including: (1) repeating the primary analysis using a binary approach to log2-ALI based on the optimal cutoff point; (2) excluding patients with cancer, malignancy, or liver disease; (3) adjusting for medication usage, including daily intake of prednisone or cortisone, current use of diabetes medication or insulin, with relevant categorization (no medication use, prednisone or cortisone only, diabetes medication or insulin only, concurrent use of prednisone or cortisone with diabetes medication or insulin); (4) considering that metabolic dysfunction-associated fatty liver disease (MAFLD), as a metabolic disease, is closely related to bone metabolism (24, 25), we included MAFLD and conducted subgroup analyses based on MAFLD (yes or no).The definition of MAFLD is provided in the **Supplementary Table S1**; (5) we incorporated the inflammatory marker C-reactive protein(CRP) to reflect the inflammatory status and analyzed the correlation between ALI and CRP to assess the reliability of ALI in reflecting inflammation in patients with T2DM. The statistical significance threshold was established at p < 0.05 (two-tailed). All statistical analyses were conducted using R Studio (version 4.2.2).

## Results

### Baseline characteristics of the population

This study included a total of 1596 middle-aged and elderly participants with T2DM, with a weighted average age of 64 years (SE = 9). Among them, 736 individuals (weighted proportion: 46%) were classified as having OP, including 360 males (47%) and 376 females (53%). Compared to the non-OP group, OP patients were likely to be older (66 (9) vs. 62 (9)) and more likely to be female (376 [53%] vs. 262 [33%]). They were also more likely to be non-Hispanic White (336 [72%] vs. 306 [66.6%]) and in the Widowed/divorced/separated status (268 [33%] vs. 209 [22%]). Additionally, significant differences between the two groups were observed in sex, BMI, ALT, blood urea nitrogen, serum uric acid, serum phosphorus, Log2-ALI, total femur, femur neck, trochanter, and intertrochanter (all p < 0.05). No significant differences were observed between the two groups in terms of PIR, smoking status, education level, physical activity, CVD, hypertension, cancer, HbA1c, AST, ALP, serum creatinine, serum calcium, total cholesterol, and triglycerides (**Table 1**).

**Table 1:** Baseline characteristics of participants with T2DM.

Characteristic	Total, N^1^ = 1596 (100%)^2^	T2DM and OP, N^1^ = 736 (46%)^2^	T2DM and non-OP, N^1^ = 860 (54%)^2^	P Value^3^
**Age (years)**	64 (9)	66 (9)	62 (9)	**<0.001**
**Sex**				**<0.001**
*female*	638 (42%)	376 (53%)	262 (33%)	
*male*	958 (58%)	360 (47%)	598 (67%)	
**Race**				**<0.001**
*Non-Hispanic White*	642 (69%)	336 (72%)	306 (66.6%)	
*Non-Hispanic Black*	333 (11%)	95 (6.1%)	238 (14.7%)	
*Mexican American*	293 (7.0%)	140 (7.5%)	153 (7.3%)	
*Other Race*	328 (13%)	165 (14.4%)	163 (11.4%)	
**PIR**				0.2
*≤1.3*	443 (17%)	219 (18.2%)	224 (15%)	
*>1.3, ≤3.5*	673 (39%)	322 (40.4%)	351 (38%)	
*>3.5*	480 (44%)	195 (41.4%)	285 (47%)	
**Smoking status**				0.9
*Current smoker*	209 (11%)	97 (11%)	112 (11.7%)	
*Former smoker*	622 (39%)	282 (39%)	340 (37.7%)	
*Never smoker*	765 (50%)	357 (50%)	408 (50.6%)	
**Education attainment**				0.12
*Less Than 9th Grade*	258 (8.0%)	129 (9.0%)	129 (7.0%)	
*9-11th Grade*	219 (10%)	96 (10%)	123 (9.0%)	
*High School Grad/GED*	402 (29%)	180 (26%)	222 (32%)	
*Some College or AA degree*	423 (30%)	208 (33%)	215 (28%)	
*College Graduate or above*	294 (23%)	123 (22%)	171 (24%)	
**Marital status**				**0.001**
*Married/cohabiting*	1,021 (68%)	427 (63%)	594 (73%)	
*Never married*	98 (5.0%)	41 (4.0%)	57 (5.0%)	
*Widowed/divorced/separated*	477 (27%)	268 (33%)	209 (22%)	
**Physical activity**				0.8
*Active*	465 (30%)	218 (30%)	247 (30%)	
*Inactive*	1,131 (70%)	518 (70%)	613 (70%)	
**CVD**	390 (24.4%)	193 (26.2%)	197 (22.9%)	0.3
**Hypertension**	1,171 (73%)	542 (73.3%)	629 (72.7%)	0.8
**Cancer**	255 (20%)	124 (22.4%)	131 (17.8%)	0.2
**HbA1c (%)**	6.94 (1.42)	6.84 (1.34)	7.03 (1.49)	0.061
**BMI (kg/m^2^)**	30.9 (5.6)	29.4 (5.3)	32.1 (5.5)	**<0.001**
**ALT (U/L)**	26 (23)	25 (13)	28 (29)	**0.026**
**AST (U/L)**	26 (15)	25 (10)	26 (17)	0.10
**ALP (U/L)**	73 (24)	74 (23)	73 (24)	0.5
**Blood urea nitrogen (mg/dL)**	16.1 (6.1)	16.7 (6.8)	15.6 (5.5)	**0.010**
**Serum Uric acid(mg/dL)**	5.70 (1.39)	5.53 (1.37)	5.84 (1.40)	**<0.001**
**Serum creatinine (µmol/L)**	0.98 (0.48)	0.98 (0.54)	0.97 (0.43)	0.7
**Serum Calcium (mg/dL)**	9.44 (0.37)	9.44 (0.39)	9.44 (0.35)	>0.9
**Serum Phosphorus (mg/dL)**	3.68 (0.58)	3.73 (0.59)	3.64 (0.56)	**0.024**
**Total Cholesterol (mg/dL)**	184 (45)	184 (45)	183 (44)	0.7
**Triglyceride (mg/dL)**	189 (137)	188 (140)	190 (135)	0.8
**Log2-ALI**	5.88 (0.74)	5.78 (0.75)	5.97 (0.73)	**<0.001**
**Total femur**	0.97 (0.16)	0.85 (0.11)	1.08 (0.12)	**<0.001**
**Femur neck**	0.80 (0.15)	0.67 (0.09)	0.90 (0.11)	**<0.001**
**Trochanter**	0.74 (0.14)	0.64 (0.10)	0.82 (0.12)	**<0.001**
**Intertrochanter**	1.16 (0.20)	1.02 (0.15)	1.28 (0.15)	**<0.001**

^1^N not Missing (unweighted);^2^Mean ± SD for continuous; n (%) for categorical;^3^t-test adapted to complex survey samples; chi-squared test with Rao & Scott's second-order correction. PIR, poverty income ratio; CVD, cardiovascular disease; HbA1c, glycated hemoglobin; ALT, alanine aminotransferase; AST, aspartate aminotransferase; ALP, alkaline phosphatase; ALI, advanced lung cancer inflammation index; OP, osteoporosis; T2DM, type 2 Diabetes mellitus. Bold values indicate p-value < 0.05.

### Association between ALI with OP and femur BMD in T2DM

The univariate analysis indicated that sex, age, race, education attainment, marital status, ALT, blood urea nitrogen, serum uric acid, serum phosphorus, and ALI were associated with OP in patients with T2DM (**Supplementary Table S2**).

The multivariable regression model shows a significant association between log2-ALI and the prevalence of OP in the population with T2DM. In the fully adjusted model (Model 3), compared to the fourth quartile of log2-ALI, the prevalence of OP significantly increased in the third to first quartiles (p for trend < 0.01), with increases of 67% (OR = 1.67; 95% CI: 1.02-2.73), 87% (OR = 1.87; 95% CI: 1.19-2.92), and 95% (OR = 1.95; 95% CI: 1.28-2.96) respectively (**Table 2**).

**Table 2:** Association between ALI and OP in T2DM.

Variable	Crude Model	Model 1	Model 2	Model3
	OR (95% CI)	OR (95% CI)	OR (95% CI)	OR (95% CI)
Log2-ALI	0.70(0.59,0.82)^** *^	0.71(0.60,0.85)^***^	0.72(0.60,0.86)^***^	0.73(0.61,0.86)^***^
Q1	2.00(1.31,3.06)^**^	2.06(1.33,3.21)^**^	1.98(1.15,2.64)^**^	1.95(1.28,2.96)^**^
Q2	2.05(1.36,3.10)^***^	1.88(1.18,2.99)^**^	1.79(1.29,3.04) ^*^	1.87(1.19, 2.92) ^**^
Q3	1.60(0.99,2.58)	1.67(0.99, 2.83)	1.64(1.00,2.68) ^*^	1.67(1.02, 2.73) ^*^
Q4	Reference	Reference	Reference	Reference
P for trend	<0.001	<0.01	<0.01	<0.01

Crude Model: Not adjusted; Model 1 adjusted for age, sex, and race; Model 2 further adjusted for smoking status, marital status, education level, PIR and physical activity on the basis of Model 1; Model 3 additionally adjusted for AST, ALT, ALP, Serum creatinine, Serum uric acid, blood urea nitrogen, Serum calcium, Serum phosphorus, HbA1c, triglycerides, total cholesterol, CVD, hypertension, and cancer on the basis of Model 2. *P < 0.05, **P < 0.01, ***P < 0.001; P < 0.05 was considered statistically significant.

Furthermore, in the fully adjusted model, log2-ALI remained significantly associated with femur BMD. Specifically, compared to the fourth quartile of log2-ALI, the first quartile showed negative correlations with total femur BMD (β = -0.06; 95% CI: -0.09 - -0.03), femoral neck BMD (β = -0.04; 95% CI: -0.07 - -0.02), trochanter BMD (β = -0.05; 95% CI: -0.07 - -0.02), and intertrochanteric BMD (β = -0.07; 95% CI: -0.10 - -0.03) (p for trend < 0.01) (**Table 3**).

**Table 3:** Association between ALI and femur BMD in T2DM.

Variable	Crude Model	Model 1	Model 2	Model3
	β (95% CI)	β (95% CI)	β (95% CI)	β (95% CI)
Total femur BMD (g/cm^2^)				
Log2-ALI	0.03(0.02,0.05)^***^	0.03(0.02,0.05)^***^	0.03(0.02,0.05)^***^	0.03(0.02,0.05)^***^
Q1	-0.06(-0.09, -0.02)^**^	-0.07(-0.09, -0.02)^***^	-0.06(-0.09, -0.03)^***^	-0.06(-0.09, -0.03)^***^
Q2	-0.04(-0.07, -0.01)^**^	-0.04(-0.06, -0.01)^**^	-0.03(-0.06, -0.01)^**^	-0.04(-0.06, -0.01)^**^
Q3	-0.02(-0.06, -0.01)	-0.03(-0.06, 0.00)^*^	-0.03(-0.06, 0.00)^*^	-0.03(-0.06, 0.00)^*^
Q4	Reference	Reference	Reference	Reference
P for trend	<0.001	<0.001	<0.001	<0.001
Femur neck BMD (g/cm^2^)				
Log2-ALI	0.03(0.02,0.04)^***^	0.02(0.01,0.03)^***^	0.02(0.01,0.03)^***^	0.02(0.01,0.03)^***^
Q1	-0.05(-0.08, -0.03)^***^	-0.05(-0.07, -0.02)^***^	-0.05(-0.07, -0.02)^***^	-0.04(-0.07, -0.02)^***^
Q2	-0.05(-0.07, -0.03)^***^	-0.03(-0.06, -0.01)^**^	-0.03(-0.05, -0.01)^**^	-0.03(-0.05, -0.01)^**^
Q3	-0.03(-0.06, 0.01)	-0.03(-0.06, 0.01)	-0.03(-0.06, 0.01)	-0.03(-0.06, 0.01)
Q4	Reference	Reference	Reference	Reference
P for trend	<0.001	<0.001	<0.001	<0.001
Trochanter BMD (g/cm^2^)				
Log2-ALI	0.03(0.02,0.04)^***^	0.03(0.02,0.04)^***^	0.03(0.02,0.04)^***^	0.03(0.02,0.04)^***^
Q1	-0.04(-0.07, -0.02)^**^	-0.05(-0.08, -0.03)^***^	-0.05(-0.08, -0.03)^***^	-0.05(-0.07, -0.02)^***^
Q2	-0.03(-0.05, -0.00)^*^	-0.03(-0.05, 0.00)^*^	-0.02(-0.05, 0.00)^*^	-0.02(-0.05, 0.00)^*^
Q3	-0.01(-0.04, 0.02)	-0.02(-0.04, 0.01)	-0.02(-0.04, 0.01)	-0.02(-0.04, 0.00)
Q4	Reference	Reference	Reference	Reference
P for trend	<0.01	<0.001	<0.001	<0.001
Intertrochanter BMD (g/cm^2^)				
Log2-ALI	0.04(0.02,0.05)^***^	0.04(0.02,0.06)^***^	0.04(0.02,0.05)^***^	0.03(0.02,0.05)^***^
Q1	-0.06(-0.10, -0.02)^**^	-0.07(-0.11, -0.04)^***^	-0.07(-0.11, -0.03)^***^	-0.07(-0.10, -0.03)^***^
Q2	-0.05(-0.08, -0.01)^**^	-0.04(-0.07, -0.01)^*^	-0.04(-0.07, -0.01)^*^	-0.04(-0.07, -0.01)^*^
Q3	-0.03(-0.08, 0.01)	-0.05(-0.09, 0.00)^*^	-0.04(-0.09, 0.00)^*^	-0.05(-0.08, -0.01)^*^
Q4	Reference	Reference	Reference	Reference
P for trend	<0.01	<0.001	<0.001	<0.01

Crude Model: Not adjusted; Model 1 adjusted for age, sex, and race; Model 2 further adjusted for smoking status, marital status, education level, PIR and physical activity on the basis of Model 1; Model 3 additionally adjusted for AST, ALT, ALP, Serum creatinine, Serum uric acid, blood urea nitrogen, Serum calcium, Serum phosphorus, HbA1c, triglycerides, total cholesterol, CVD, hypertension, and cancer on the basis of Model 2. *P < 0.05, **P < 0.01, ***P < 0.001; P < 0.05 was considered statistically significant.

### Nonlinear relationship and ROC curve

**Figure 2:**
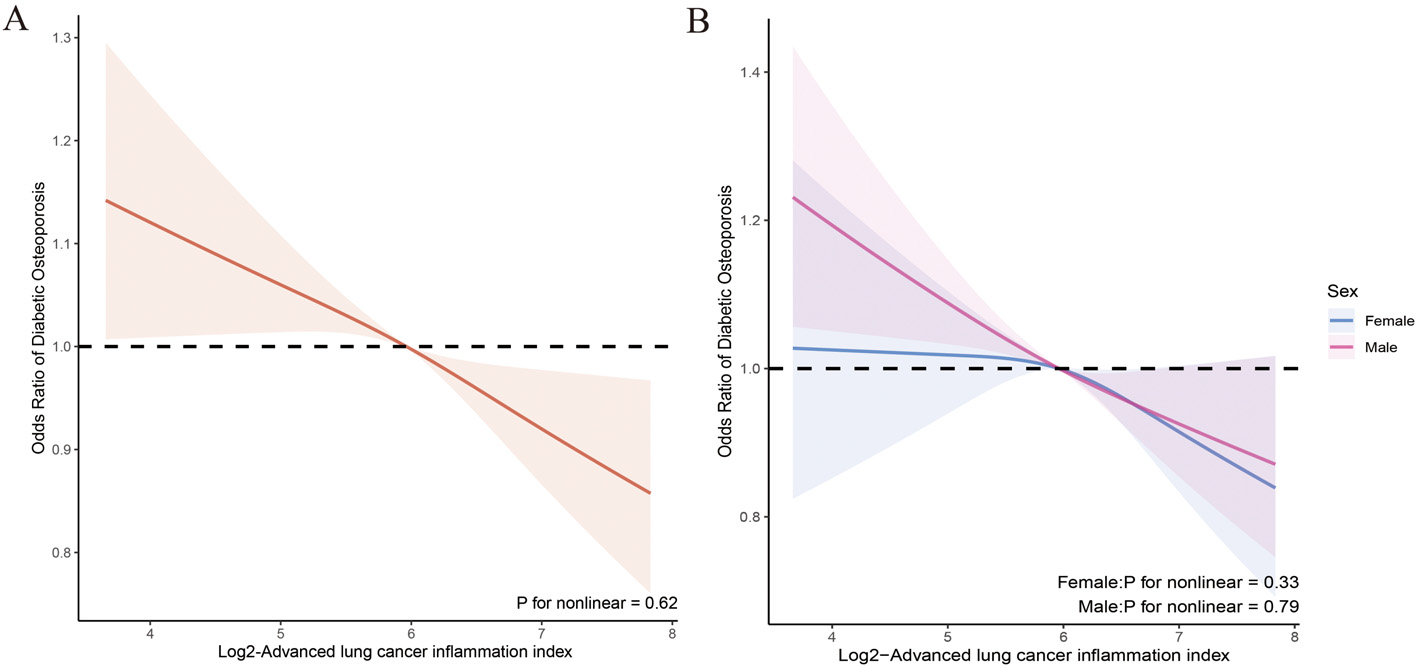
Relationship between Log2-ALI and the prevalence of OP in patients with T2DM. **(A)** Overall population; **(B)** Stratified population by sex.

**Figure 3:**
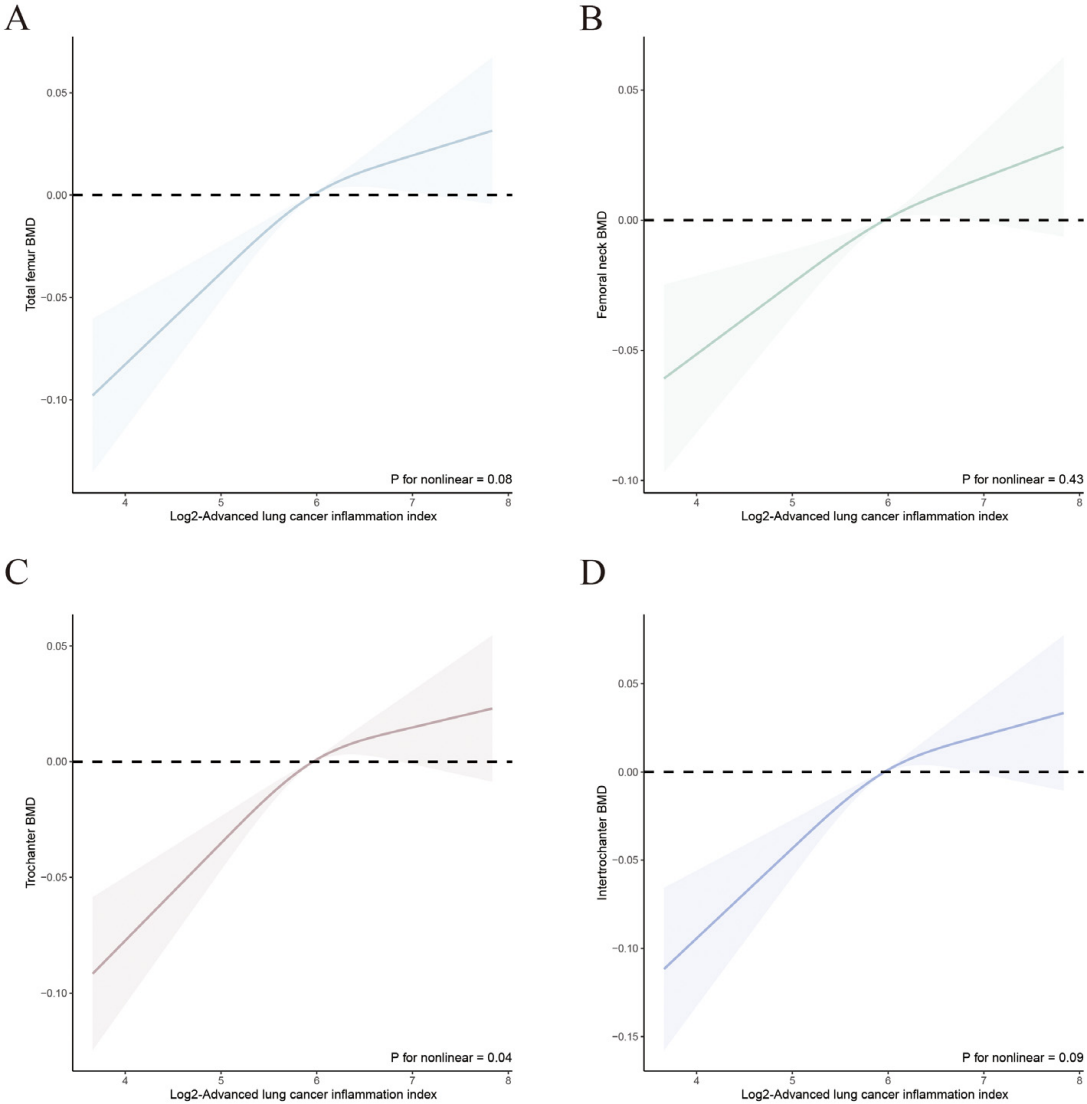
Relationship between Log2-ALI and femur BMD in T2DM. **(A)** Total femur BMD; **(B)** Femur neck BMD; **(C)** Trochanter BMD; **(D)** Intertrochanter BMD.

We evaluated the dose-response relationship between ALI and OP, as well as femur BMD in patients with T2DM based on Model 3. RCS analysis indicated a linear dose-response relationship between log2-ALI and OP in the overall population (p for nonlinear = 0.62). Stratified analysis by gender showed consistent trends with the overall population (all p for nonlinear > 0.05) (**Figure 2**). Regarding femur BMD, a nonlinear relationship was observed between log2-ALI and trochanter BMD (p for nonlinear = 0.04), while linear relationships were found with total femur BMD, femur neck BMD, and intertrochanter BMD (all p for nonlinear > 0.05) (**Figure 3**).

**Figure 4:**
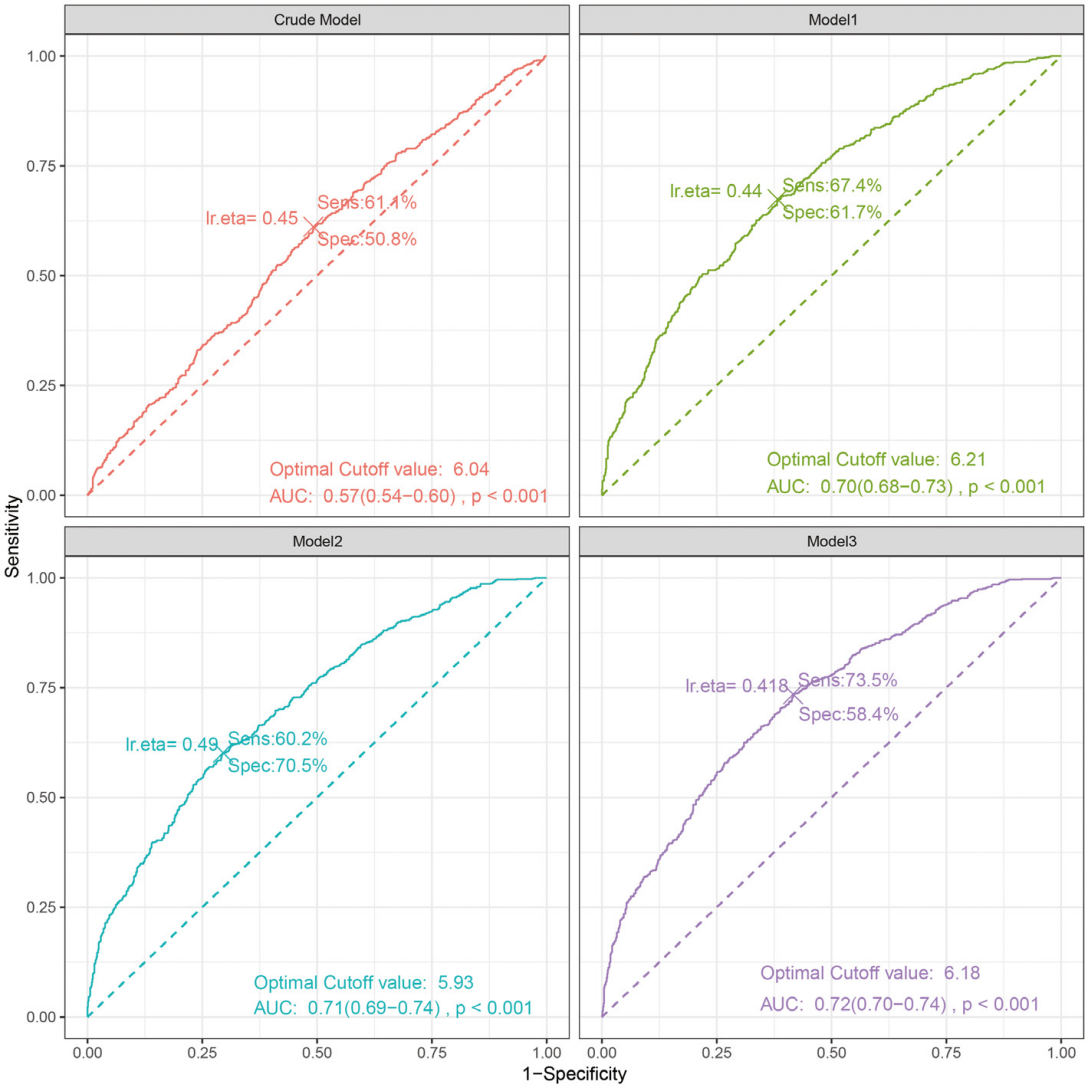
The ROC curve between Log2-ALI (continuous) and OP in T2DM. Sens, sensibility; Spec, specificity; AUC, area under the curve.

The ROC curve indicated that the area under the curve (AUC) for predicting the ROC curve when Log2-ALI was considered as a continuous variable was 0.57 (95% CI: 0.54-0.60, p < 0.001). The optimal cutoff value was 6.04, with corresponding sensitivity and specificity of 61.1% and 50.8%, respectively. Furthermore, the predictive ROC curve areas for models 1, 2, and 3 were all statistically significant, with an AUC of 0.72 (95% CI: 0.70–0.74, p < 0.001) in the fully adjusted model (Model 3) (**Figure 4**).

### Subgroup and sensitivity analysis

**Figure 5:**
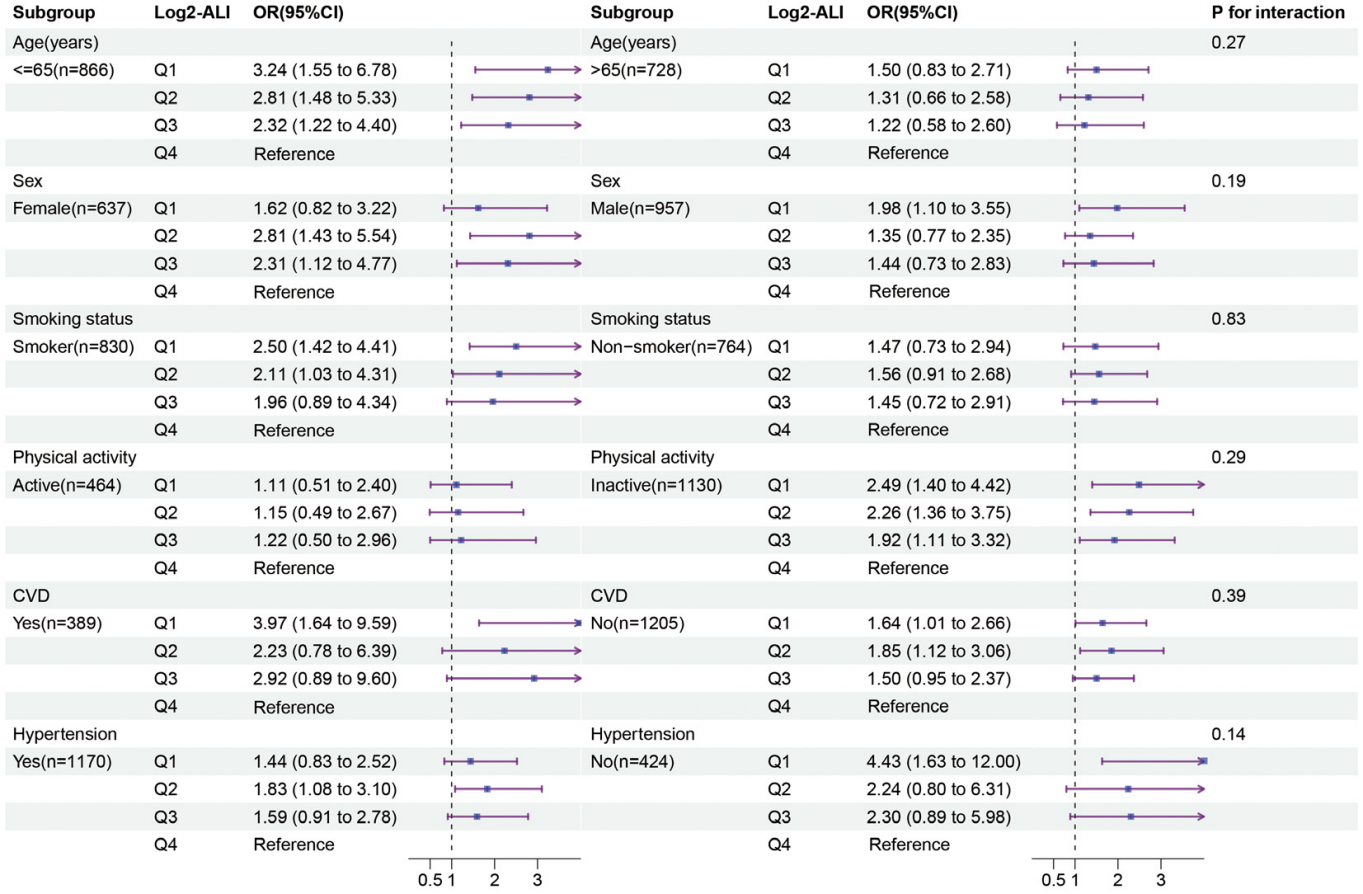
Relationship between ALI and OP in different subgroups of patients with T2DM.

We examined the potential differences in the association between ALI and OP among patients with T2DM based on age, sex, smoking status, physical activity, CVD, and hypertension subgroups. The results indicated that, compared to the fourth quartile of Log2-ALI, the first quartile was more strongly associated with the prevalence of OP in individuals under 60 years old (OR = 3.24; 95% CI: 1.55–6.78), smokers (OR = 2.50; 95% CI: 1.42–4.41), and those with low physical activity (OR = 2.49; 95% CI: 1.40–4.42) (**Figure 5**). No significant differences were found in terms of sex, CVD, and hypertension. Additionally, subgroup analysis for MAFLD showed that, compared to the fourth quartile of Log2-ALI, the first quartile exhibited a stronger association with the prevalence of OP in MAFLD patients (OR = 5.76; 95% CI: 1.11–29.9) (**Supplementary Table S3**). Finally, no interactions were observed in any of the above subgroups (all p for interaction > 0.05).

Based on the optimal cutoff value of Log2-ALI determined by the ROC curve (6.04), Log2-ALI was divided into two groups (high: >6.04, low: ≤6.04), and weighted logistic regression was used to analyze the association between Log2-ALI and OP in T2DM again. The results indicated that in the fully adjusted model, the low Log2-ALI group was consistently positively associated with the prevalence of OP compared to the high Log2-ALI group (OR = 1.62; 95% CI: 1.20 – 2.19) (**Supplementary Table S4**). Furthermore, compared to the high Log2-ALI group, the low Log2-ALI group showed significant negative correlations with total femur BMD (β = -0.04; 95% CI: -0.06 – -0.02), femoral neck BMD (β = -0.02; 95% CI: -0.04 – 0.00), trochanteric BMD (β = -0.03; 95% CI: -0.05 – -0.01), and intertrochanteric BMD (β = -0.04; 95% CI: -0.06 – -0.01) (**Supplementary Table S5**).

After excluding participants with cancer, malignancies, or liver disease, a total of 1,233 participants remained. We then reanalyzed the association between Log2-ALI and OP in individuals with T2DM. The results we obtained were stable; specifically, in the fully adjusted model, the prevalence of OP in the first quartile of Log2-ALI was positively correlated with that of participants in the fourth quartile (OR = 2.03; 95% CI: 1.23–3.33) (**Supplementary Table S6**). We also observed consistent directional associations between Log-ALI and femur BMD in line with previous analyses (**Supplementary Table S7**).

Certainly, we also adjusted for medication use, and the association between Log2-ALI and OP, as well as between Log2-ALI and femur BMD, remained consistent. In the fully adjusted model, compared to participants in the fourth quartile, the Log2-ALI in the second quartile showed a positive correlation with the prevalence of OP (OR = 1.76; 95% CI: 1.08 – 2.86) (**Supplementary Table S8**). Furthermore, there were significant negative correlations between Log2-ALI in the first quartile and total femur BMD (β = -0.05; 95% CI: -0.08 – -0.02), femoral neck BMD (β = -0.04; 95% CI: -0.06 – -0.01), trochanteric BMD (β = -0.04; 95% CI: -0.07 – -0.01), and intertrochanteric BMD (β = -0.06; 95% CI: -0.10 – -0.02) compared to participants in the fourth quartile (**Supplementary Table S9**).

Finally, we included CRP in the analysis, with 878 participants remaining. The baseline characteristics of the participants are shown in **Supplementary Table S10**, where a significant difference in CRP was observed between the OP and non-OP groups (p < 0.05). Additionally, we performed a correlation analysis between Log2-ALI and CRP using weighted logistic regression. The results indicated that in the fully adjusted model, there was a negative correlation between Log2-ALI and CRP (β = -0.13; 95% CI: -0.22, -0.05) (**Supplementary Table S11**). Overall, the results of the sensitivity analysis were stable.

## Discussion

T2DM and OP are both metabolic diseases with significant global prevalence. In this study, we focused on middle-aged and older patients with T2DM in the United States and found that low levels of ALI are associated with the prevalence of osteoporosis and low femur BMD among T2DM patients. This association remains significant even after accounting for relevant confounding variables. Additionally, further RCS analysis revealed a linear relationship between ALI levels and the prevalence of OP in T2DM patients, which is consistent across both genders. Through ROC curve analysis, the optimal cutoff value was determined to be 6.04. Various subgroup analyses and sensitivity assessments further reinforced the robustness of our conclusions. In summary, evaluating ALI levels could aid in identifying the prevalence of osteoporosis among T2DM patients in the United States.

The occurrence of OP is closely related to significant alterations in the bone remodeling process due to the imbalance between osteoclasts and osteoblasts (26), with oxidative-reductive status and inflammation playing crucial roles in osteoclast and osteoblast generation (27). Excessive production of ROS and elevated levels of pro-inflammatory cytokines can impair bone cell function, negatively impacting osteoblast proliferation, differentiation, and maturation, while accelerating the generation of osteoclasts, leading to changes in bone structure and loss of bone mass (28, 29). In comparison to healthy individuals, inflammatory cytokine levels are higher in diabetic populations, potentially indicating a chronic inflammatory state (30, 31). Additionally, hyperglycemic conditions can trigger the release of pro-inflammatory cytokines, leading to ROS production (32, 33), directly affecting osteoblast differentiation and survival. It is noteworthy that prolonged hyperglycemia, inflammation, and oxidative stress contribute to elevated levels of advanced glycation end products (AGEs) in T2DM. On one hand, AGEs accumulation leads to decreased bone turnover rates and compromised bone strength (34), while on the other hand, AGEs inhibit crucial endoplasmic reticulum functions essential for osteoblast differentiation and activity, and increase osteoblast apoptosis (35, 36). Activation of AGEs receptors in human osteoblasts exacerbates the generation of inflammatory cytokines and ROS, perpetuating a vicious cycle between inflammation and bone resorption (37). Furthermore, inflammation may contribute to another risk factors for OP, malnutrition (38, 39), as it reduces the effective utilization of dietary proteins and enhances the breakdown metabolism of key cellular proteins (40). Additionally, individuals with T2DM may incur additional risks of nutritional deficiencies by excessively restricting their dietary intake to manage blood sugar levels (41). Hence, OP in T2DM patients should be given greater attention.

ALI have been used in the assessment of various chronic diseases, such as rheumatoid arthritis and the prognosis of patients with hypertension (19, 42). ALI has been used to assess the prognosis of various chronic diseases, such as rheumatoid arthritis and hypertension in patients. Some studies have indicated a significant correlation between elevated ALI levels and reduced all-cause mortality in patients with T2DM (43), further supporting our research. Previous studies have shown that T2DM patients are more susceptible to OP (44, 45), which significantly increases the risk of fractures, leading to higher rates of disability, mortality, and economic costs, especially among the elderly. Our research indicates that ALI is associated with osteoporosis in T2DM patients. Firstly, the ALI score comprises BMI, serum albumin levels, and NLR, where BMI and albumin levels reflect nutritional status, and NLR reflects inflammatory status, making ALI a comprehensive assessment of nutrition and inflammation. Neutrophils serve as frontline innate immune defenders, crucially recruiting immune cells to sites of inflammation (46). Conversely, lymphocytes primarily mediate adaptive immunity, exerting regulatory or protective functions, with low lymphocyte counts typically indicating poor conditions (47). Therefore, NLR reflects two distinct immune pathways and may better reflect inflammatory status compared to individual inflammatory markers. Studies have revealed a strong correlation between elevated NLR and OP (48, 49), as evidenced by meta-analyses (50), which aligns with our research findings. Reports indicate that inflammation induced by lymphocyte dysfunction triggers a series of inflammatory cytokines and chemokines, resulting in the aggregation of neutrophils and macrophages, thereby disrupting the delicate balance of bone formation (51, 52). Additionally, the excessive activation of neutrophils releases reactive oxygen species and enhances the receptor activator of nuclear factor kappa-B ligand signaling pathway, accelerating the apoptosis of osteoblasts and promoting the generation of osteoclasts (53, 54).

Currently, a substantial body of research indicates a close association between low serum albumin levels and the development of osteoporosis (55, 56), Some studies report a direct correlation between hypoalbuminemia and the activation of nuclear factor kappa-B (57), a process that involves the inhibition of osteoblasts and the activation of osteoclasts (58). Furthermore, hypoalbuminemia can lead to reduced synthesis of insulin-like growth factor-1, thereby affecting factors such as the quantity and activity of osteoblasts and bone remodeling (55). BMI, as a modifiable risk factor for T2DM and OP, has been studied in relation to increased risk of OP with lower body weight (59). A study in a Korean population indicated that men (BMI: 25.0-29.9 kg/m^2^) and women (BMI: ≥30 kg/m^2^) had a more than 60% lower prevalence of OP compared to the reference group (BMI: 21.0-22.9 kg/m^2^); however, it’s crucial to note the risks linked with obesity, as it is associated with higher mortality rates in T2DM populations (60). The increase in BMI associated with T2DM may result in elevated pro-inflammatory cytokines and circulating lipid factors, which can exacerbate mechanical loading on skeletal sites, ultimately promoting bone resorption (61). In conclusion, the relationship between BMI and T2DM and OP is complex and significant. Future prospective studies will help determine appropriate BMI ranges to guide the more effective application of ALI biomarkers and more precise health management measures.

In our further subgroup analysis, we found that ALI in the MAFLD subgroup is more closely associated with the prevalence of OP. MAFLD is one of the major complications of T2DM, involving hepatic lipid accumulation, inflammation, and liver fibrosis (62, 63). In MAFLD, as liver lipid deposition increases, excessive accumulation leads to the production of lipotoxic substances, which in turn triggers oxidative stress, mitochondrial dysfunction, and endoplasmic reticulum stress (64). ROS are the primary type of free radicals involved in the disruption of bone remodeling. When the mechanisms of ROS production are enhanced and overcome the antioxidant system, their excessive generation leads to redox imbalance, resulting in increased differentiation of osteoclasts, further supporting increased bone loss and promoting the development of OP (65, 66). Additionally, all our study participants were T2DM patients, which may further exacerbate oxidative stress. However, it is necessary to conduct further research in the future to explore the underlying mechanisms behind these complex associations.

Our study boasts several strengths. Firstly, it draws from a representative national sample, offering extensive scale.

Secondly, our findings provide valuable insights for clinical applications, alerting healthcare practitioners to the potential complications of OP or bone loss in patients with T2DM who have low ALI levels. Furthermore, ALI serves as a comprehensive indicator reflecting nutrition and inflammation, emphasizing the necessity for timely nutritional and anti-inflammatory interventions. Thirdly, the ALI calculation is simple and cost-effective, making it a viable biomarker for the early screening of OP in elderly patients with T2DM.

We also acknowledge some limitations. First, our study is cross-sectional, which prevents us from inferring causality. Second, some covariate information relies on self-reported questionnaire assessments, which may introduce recall bias. Third, although we controlled for key demographic factors, laboratory measurements, behavioral risk factors, and various chronic diseases, we cannot completely rule out the influence of other potential confounding variables. Lastly, our study was conducted in a representative sample from the United States, which limits the generalizability of the findings. In future research, we recommend conducting longitudinal studies to regularly track changes in ALI levels and bone density in patients with T2DM, which will help determine the causal relationship between ALI and OP. Similarly, intervention studies involving nutritional interventions and anti-inflammatory treatments to evaluate their effects on the prevalence of OP in T2DM will provide more direct evidence regarding the importance of nutrition and inflammation in the development of OP among T2DM patients. Moreover, considering multicenter studies may help improve the generalizability of the results.

## Conclusion

Our study found that low levels of ALI are independently associated with an increased prevalence of OP in middle-aged and elderly patients with T2DM in the United States. This suggests that maintaining good nutritional status and anti-inflammatory treatment may have a positive impact on bone health. Additionally, ALI can serve as a useful biomarker for early screening of OP in clinical practice among middle-aged and elderly individuals with T2DM.

## Data Availability

The original contributions presented in the study are included in the article/**Supplementary Material**. Further inquiries can be directed to the corresponding author.
