# Supplementary material for: Association between advanced lung cancer inflammation index and osteoporosis in patients with type 2 diabetes mellitus: evidence from NHANES
Source: Front Endocrinol (Lausanne). 2024 Nov 25;15:1421696. doi: 10.3389/fendo.2024.1421696 (PMC11625538; doi:10.3389/fendo.2024.1421696)
Supplement: Supplementary file 1 [file DataSheet1.docx]

Table S1 The definition of metabolic dysfunction-associated fatty liver disease

| According to the 2020 international expert consensus statement [1], a positive diagnosis of metabolic dysfunction-associated fatty liver disease (MAFLD) is based on Vibration-Controlled Transient Elastography (VCTE) evidence of fat accumulation in the liver (hepatic steatosis) in addition to one of the following three criteria, namely overweight/obesity, presence of type 2 diabetes mellitus (T2DM), or evidence of metabolic dysregulation.  Metabolic dysregulation exhibits the following characteristics: 1) waist circumference ≥102/88 cm in Caucasian men and women; 2) blood pressure ≥130/85 mmHg or specific drug treatment; 3) plasma triglycerides ≥150 mg/dl or specific drug treatment; 4) plasma High-Density Lipoprotein(HDL)-cholesterol <40 mg/dl for men and <50 mg/dl for women or specific drug treatment; 5) prediabetes (fasting glucose levels 100 to 125 mg/dl, or glycated hemoglobin (HbA1c) 5.7% to 6.4%; 6) Homeostasis Model Assessment of Insulin Resistance (HOMA-IR) score ≥2.5; 7) plasma high-sensitivity C-reactive protein level >2 mg/L. |
| --- |

1.Eslam M, Newsome PN, Sarin SK, Anstee QM, Targher G, Romero-Gomez M, Zelber-Sagi S, Wai-Sun Wong V, Dufour JF, Schattenberg JM et al: A new definition for metabolic dysfunction-associated fatty liver disease: An international expert consensus statement. J Hepatol 2020, 73(1):202-209.

Table S2 Univariate Analysis Results of OP in T2DM

| **Variable** | **OR (95%CI)** | **P-value** |
| --- | --- | --- |
| **Age (years)** | 1.05 (1.04,1.07) | **<0.001** |
| **Sex** |  |  |
| Female | Reference |  |
| Male | 0.44 (0.32~0.61) | **<0.001** |
| **Race** |  |  |
| Non-Hispanic White | Reference |  |
| Non-Hispanic Black | 0.39 (0.28~0.53) | **<0.001** |
| Other Race | 1.17 (0.79~1.73) | 0.436 |
| Mexican American | 0.95 (0.69~1.33) | 0.773 |
| **Education attainment** |  |  |
| Less Than 9th Grade | Reference |  |
| 9-11th Grade | 0.88 (0.54~1.43) | 0.604 |
| High School Grad/GED | 0.65 (0.43~0.99) | **0.045** |
| Some College or AA degree | 0.97 (0.63,1.51) | 0.894 |
| College Graduate or above | 0.72 (0.44,1.16) | 0.172 |
| **Marital status** |  |  |
| Married/cohabiting | Reference |  |
| Never married | 1.05 (0.63~1.73) | 0.852 |
| Widowed/divorced/separated | 1.72 (1.24~2.38) | **0.001** |
| **PIR** |  |  |
| >3.5 | Reference |  |
| ≤1.3 | 1.37(0.96,1.95) | 0.079 |
| >1.3, ≤3.5 | 1.21(0.88,1.67) | 0.245 |
| **Smoking status** |  |  |
| Current smoker | Reference |  |
| Former smoker | 1.10(0.73,1.66) | 0.653 |
| Never smoker | 1.04(0.71,1.52) | 0.838 |
| **Physical activity** |  |  |
| Active | Reference |  |
| Inactive | 0.96(0.70,1.32) | 0.797 |
| **CVD** |  |  |
| Yes | Reference |  |
| No | 0.84(0.58,1.21) | 0.343 |
| **Hypertension** |  |  |
| Yes | Reference |  |
| No | 0.97(0.73,1.30) | 0.842 |
| **Cancer** |  |  |
| Yes | Reference |  |
| No | 0.75(0.48,1.18) | 0.213 |
| **HbA1c (%)** | 0.91(0.83,1.01) | 0.064 |
| **ALT（U/L）** | 0.99(0.98,1.00) | **0.033** |
| **AST（U/L）** | 0.99(0.98,1.00) | 0.107 |
| **ALP（U/L）** | 1.00(1.00,1.01) | 0.550 |
| **Blood urea nitrogen (mg/dL)** | 1.03(1.01,1.05) | **0.013** |
| **Serum uric acid (mg/dL)** | 0.85(0.77,0.93) | **0.001** |
| **Serum creatinine** **(mg/dl)** | 1.04(0.84,1.28) | 0.732 |
| **Serum Calcium (mg/dL)** | 1.00(0.67,1.50) | 0.988 |
| **Serum Phosphorus (mg/dL)** | 1.31(1.04,1.66) | **0.024** |
| **Total Cholesterol** **(mg/dL)** | 1.00(1.00,1.00) | 0.683 |
| **Triglyceride (mg/dL)** | 1.00(1.00,1.00) | 0.827 |
| **Log2-****ALI** | 0.70(0.59,0.82) | **<0.001** |

95% CI, 95% confidence interval; OR, odds ratio; PIR, poverty income ratio; CVD, cardiovascular disease; HbA1c, glycated hemoglobin; ALT, alanine aminotransferase; AST, aspartate aminotransferase; ALP, alkaline phosphatase; ALI, advanced lung cancer inflammation index. Bold values indicate p-value < 0.05.

Table S3 Relationship between ALI and OP in MAFLD subgroups of patients with T2DM

| **Characteristic** | **Log2-ALI** | **OR (95% CI)** | **p-value** | ***P* for interaction** |
| --- | --- | --- | --- | --- |
| **MAFLD** |  |  |  | 0.54 |
| Yes |  |  |  |  |
|  | Quartile 1 | 2.29(0.38, 13.9) | 0.30 |  |
|  | Quartile 2 | 5.76(1.11, 29.9) | 0.04 |  |
|  | Quartile 3 | 2.87(0.61, 13.5) | 0.20 |  |
|  | Quartile 4 | — |  |  |
| No |  |  |  |  |
|  | Quartile 1 | 0.24(0.01, 11.1) | 0.30 |  |
|  | Quartile 2 | 0.62(0.05, 7.76) | 0.60 |  |
|  | Quartile 3 | 1.54(0.17, 13.9) | 0.60 |  |
|  | Quartile 4 | — |  |  |

MAFLD, Metabolic-associated Fatty Liver Disease; OR, Odds Ratio; CI, Confidence Interval.

Table S4 Association between ALI and OP in T2DM (Classified according to the optimal cutoff value)

| Variable | Crude Model | Model 1 | Model 2 | Model3 |
| --- | --- | --- | --- | --- |
|  | OR (95% CI) | OR (95% CI) | OR (95% CI) | OR (95% CI) |
| Log2-ALI | 0.70(0.59,0.82) **^** *^** | 0.71(0.60,0.85) **^***^** | 0.72(0.60,0.86) **^***^** | 0.73(0.61,0.86) **^***^** |
| ≤6.04 | 1.74(1.33,2.28) **^***^** | 1.62(1.22,2.15) **^**^** | 1.59(1.19,2.11) **^**^** | 1.62(1.20,2.19) **^**^** |
| >6.04 | Reference | Reference | Reference | Reference |
| P for trend | <0.001 | <0.01 | <0.01 | <0.01 |

95% CI, 95% confidence interval; OR, odds ratio; ALI, advanced lung cancer inflammation index. Crude Model: Not adjusted; Model 1 adjusted for age, sex, and race; Model 2 further adjusted for smoking status, marital status, education level, PIR, and physical activity on the basis of Model 1; Model 3 additionally adjusted for AST, ALT, ALP, Serum creatinine, Serum uric acid, blood urea nitrogen, Serum calcium, Serum phosphorus, HbA1c, triglycerides, total cholesterol, CVD, hypertension, and cancer. * P < 0.05, ** P < 0.01, *** P < 0.001; P < 0.05 was considered statistically significant.

Table S5 Association between ALI and femur BMD in T2DM (Classified according to the optimal cutoff value)

| Variable | Crude Model | Model 1 | Model 2 | Model3 |
| --- | --- | --- | --- | --- |
|  | β (95% CI) | β (95% CI) | β (95% CI) | β (95% CI) |
| Total femur BMD (g/cm^2^) |  |  |  |  |
| Log2-ALI | 0.03(0.02,0.05) **^***^** | 0.03(0.02,0.05) **^***^** | 0.03(0.02,0.05) **^***^** | 0.03(0.02,0.05) **^***^** |
| ≤6.04 | -0.04(-0.07, -0.02) **^***^** | -0.04(-0.06, -0.02) **^***^** | -0.04(-0.06, -0.02) **^***^** | -0.04(-0.06, -0.02) **^***^** |
| >6.04 | Reference | Reference | Reference | Reference |
| P for trend | <0.001 | <0.001 | <0.001 | <0.001 |
| Femur neck BMD (g/cm^2^) |  |  |  |  |
| Log2-ALI | 0.03(0.02,0.04) **^***^** | 0.02(0.01,0.03) **^***^** | 0.02(0.01,0.03) **^***^** | 0.02(0.01,0.03) **^***^** |
| ≤6.04 | -0.04(-0.06, -0.02) **^***^** | -0.03(-0.04, -0.01) **^**^** | -0.02(-0.04, -0.01) **^*^** | -0.02(-0.04, 0.00) **^*^** |
| >6.04 | Reference | Reference | Reference | Reference |
| P for trend | <0.001 | <0.001 | <0.001 | <0.001 |
| Trochanter BMD (g/cm^2^) |  |  |  |  |
| Log2-ALI | 0.03(0.02,0.04) **^***^** | 0.03(0.02,0.04) **^***^** | 0.03(0.02,0.04) **^***^** | 0.03(0.02,0.04) **^***^** |
| ≤6.04 | -0.05(-0.07, -0.02) **^**^** | -0.03(-0.05, -0.02) **^***^** | -0.03(-0.05, -0.02) **^***^** | -0.03(-0.05, -0.01) **^***^** |
| >6.04 | Reference | Reference | Reference | Reference |
| P for trend | <0.01 | <0.001 | <0.001 | <0.001 |
| Intertrochanter BMD (g/cm^2^) |  |  |  |  |
| Log2-ALI | 0.04(0.02,0.05) **^***^** | 0.04(0.02,0.06) **^***^** | 0.04(0.02,0.05) **^***^** | 0.03(0.02,0.05) **^***^** |
| ≤6.04 | -0.04(-0.05, -0.02) **^***^** | -0.04(-0.07, -0.02) **^**^** | -0.04(-0.06, -0.01) **^**^** | -0.04(-0.06, -0.01) **^**^** |
| >6.04 | Reference | Reference | Reference | Reference |
| P for trend | <0.01 | <0.001 | <0.001 | <0.01 |

95% CI, 95% confidence interval; OR, odds ratio; ALI, advanced lung cancer inflammation index. Crude Model: Not adjusted; Model 1 adjusted for age, sex, and race; Model 2 further adjusted for smoking status, marital status, education level, PIR, and physical activity on the basis of Model 1; Model 3 additionally adjusted for AST, ALT, ALP, Serum creatinine, Serum uric acid, blood urea nitrogen, Serum calcium, Serum phosphorus, HbA1c, triglycerides, total cholesterol, CVD, hypertension, and cancer. * P < 0.05, ** P < 0.01, *** P < 0.001; P < 0.05 was considered statistically significant.

Table S6 Association between ALI and OP in T2DM (Participants with cancer, malignancies, or liver disease were excluded)

| Variable | Crude Model | Model 1 | Model 2 | Model3 |
| --- | --- | --- | --- | --- |
|  | OR (95% CI) | OR (95% CI) | OR (95% CI) | OR (95% CI) |
| Log2-ALI | 0.64(0.52,0.79) **^** *^** | 0.65(0.51,0.83) **^***^** | 0.65(0.51,0.84) **^**^** | 0.65(0.50,0.84) **^**^** |
| Q1 | 2.06(1.34,3.17) **^**^** | 2.13(1.34,3.39) **^**^** | 2.05(1.29,3.25) **^**^** | 2.03(1.23,3.33) **^**^** |
| Q2 | 2.02(1.19,3.42) **^**^** | 1.84(0.98,3.48) | 1.74(0.94,3.25) | 1.80(0.97, 3.35) |
| Q3 | 1.32(0.83,2.11) | 1.31(0.78, 2.22) | 1.28(0.76,2.14) | 1.24(0.74, 2.06) |
| Q4 | Reference | Reference | Reference | Reference |
| P for trend | <0.001 | <0.01 | <0.01 | <0.01 |

95% CI, 95% confidence interval; OR, odds ratio; ALI, advanced lung cancer inflammation index. Crude Model: Not adjusted; Model 1 adjusted for age, sex, and race; Model 2 further adjusted for smoking status, marital status, education level, PIR, and physical activity on the basis of Model 1; Model 3 additionally adjusted for AST, ALT, ALP, Serum creatinine, Serum uric acid, blood urea nitrogen, Serum calcium, Serum phosphorus, HbA1c, triglycerides, total cholesterol, CVD, and hypertension. * P < 0.05, ** P < 0.01, *** P < 0.001; P < 0.05 was considered statistically significant.

Table S7 Association between ALI and femur BMD in T2DM (Participants with cancer, malignancies, or liver disease were excluded)

| Variable | Crude Model | Model 1 | Model 2 | Model3 |
| --- | --- | --- | --- | --- |
|  | β (95% CI) | β (95% CI) | β (95% CI) | β (95% CI) |
| Total femur BMD (g/cm^2^) |  |  |  |  |
| Log2-ALI | 0.04(0.02,0.06) **^***^** | 0.04(0.02,0.06) **^***^** | 0.04(0.02,0.06) **^***^** | 0.04(0.02,0.06) **^***^** |
| Q1 | -0.06(-0.10, -0.03) **^***^** | -0.07(-0.11, -0.04) **^***^** | -0.07(-0.10, -0.04) **^***^** | -0.07(-0.10, -0.04) **^***^** |
| Q2 | -0.05(-0.09, -0.01) **^**^** | -0.04(-0.08, -0.01) **^*^** | -0.04(-0.08, -0.01) **^**^** | -0.05(-0.08, -0.01) **^**^** |
| Q3 | -0.01(-0.05, 0.03) | -0.01(-0.05, 0.02) | -0.02(-0.05, 0.01) | -0.02(-0.05, 0.01) |
| Q4 | Reference | Reference | Reference | Reference |
| P for trend | <0.001 | <0.001 | <0.001 | <0.001 |
| Femur neck BMD (g/cm^2^) |  |  |  |  |
| Log2-ALI | 0.04(0.02,0.05) **^***^** | 0.03(0.01,0.04) **^***^** | 0.03(0.01,0.04) **^***^** | 0.03(0.01,0.04) **^***^** |
| Q1 | -0.06(-0.09, -0.03) **^***^** | -0.05(-0.08, -0.02) **^**^** | -0.05(-0.08, -0.02) **^**^** | -0.04(-0.07, -0.01) **^**^** |
| Q2 | -0.06(-0.09, -0.03) **^***^** | -0.04(-0.07, -0.01) **^*^** | -0.04(-0.07, -0.01) **^*^** | -0.04(-0.07, -0.01) **^*^** |
| Q3 | -0.01(-0.05, 0.02) | -0.01(-0.04, 0.02) | -0.01(-0.04, 0.02) | -0.01(-0.04, 0.02) |
| Q4 | Reference | Reference | Reference | Reference |
| P for trend | <0.001 | <0.001 | <0.001 | <0.01 |
| Trochanter BMD (g/cm^2^) |  |  |  |  |
| Log2-ALI | 0.03(0.02,0.05) **^***^** | 0.03(0.02,0.05) **^***^** | 0.03(0.02,0.05) **^***^** | 0.03(0.02,0.05) **^***^** |
| Q1 | -0.05(-0.08, -0.02) **^**^** | -0.06(-0.09, -0.03) **^***^** | -0.06(-0.08, -0.03) **^***^** | -0.05(-0.08, -0.03) **^***^** |
| Q2 | -0.03(-0.06, -0.00) | -0.03(-0.06, 0.00) | -0.03(-0.06, 0.00) | -0.03(-0.06, 0.00) **^*^** |
| Q3 | 0.00(-0.03, 0.03) | -0.01(-0.03, 0.02) | -0.01(-0.04, 0.02) | -0.01(-0.04, 0.02) |
| Q4 | Reference | Reference | Reference | Reference |
| P for trend | <0.001 | <0.001 | <0.001 | <0.001 |
| Intertrochanter BMD (g/cm^2^) |  |  |  |  |
| Log2-ALI | 0.05(0.03,0.07) **^***^** | 0.05(0.03,0.07) **^***^** | 0.05(0.03,0.07) **^***^** | 0.05(0.03,0.07) **^***^** |
| Q1 | -0.07(-0.11, -0.03) **^***^** | -0.08(-0.12, -0.05) **^***^** | -0.08(-0.12, -0.04) **^***^** | -0.08(-0.12, -0.04) **^***^** |
| Q2 | -0.06(-0.10, -0.02) **^**^** | -0.06(-0.10, -0.02) **^**^** | -0.06(-0.09, -0.02) **^**^** | -0.06(-0.10, -0.02) **^**^** |
| Q3 | -0.02(-0.06, 0.02) | -0.02(-0.06, 0.01) | -0.02(-0.06, 0.01) | -0.03(-0.06, -0.01) |
| Q4 | Reference | Reference | Reference | Reference |
| P for trend | <0.001 | <0.001 | <0.001 | <0.001 |

95% CI, 95% confidence interval; OR, odds ratio; ALI, advanced lung cancer inflammation index. Crude Model: Not adjusted; Model 1 adjusted for age, sex, and race; Model 2 further adjusted for smoking status, marital status, education level, PIR, and physical activity on the basis of Model 1; Model 3 additionally adjusted for AST, ALT, ALP, Serum creatinine, Serum uric acid, blood urea nitrogen, Serum calcium, Serum phosphorus, HbA1c, triglycerides, total cholesterol, CVD, and hypertension. * P < 0.05, ** P < 0.01, *** P < 0.001; P < 0.05 was considered statistically significant.

Table S8 Association between ALI and OP in T2DM (The medication usage was adjusted)

| Variable | Crude Model | Model 1 | Model 2 | Model3 |
| --- | --- | --- | --- | --- |
|  | OR (95% CI) | OR (95% CI) | OR (95% CI) | OR (95% CI) |
| Log2-ALI | 0.76(0.64,0.90) ** | 0.75(0.63,0.91) ** | 0.77(0.65,0.93) ** | 0.76(0.63,0.92) ** |
| Q1 | 1.58(0.98,2.56) | 1.69(1.03,2.78) * | 1.59(0.97,2.60) | 1.63(0.97,2.72) |
| Q2 | 1.88(1.22,2.90) ** | 1.73(1.05,2.85) * | 1.65(1.01,2.68) * | 1.76(1.08, 2.86) * |
| Q3 | 1.49(0.87,2.55) | 1.55(0.84,2.86) | 1.52(0.85,2.73) | 1.56(0.86, 2.82) |
| Q4 | Reference | Reference | Reference | Reference |
| P for trend | 0.02 | 0.03 | 0.05 | 0.05 |

95% CI, 95% confidence interval; OR, odds ratio; ALI, advanced lung cancer inflammation index. Crude Model: Not adjusted; Model 1 adjusted for age, sex, and race; Model 2 further adjusted for smoking status, marital status, education level, PIR, physical activity, and medication usage on the basis of Model 1; Model 3 additionally adjusted for AST, ALT, ALP, Serum creatinine, Serum uric acid, blood urea nitrogen, Serum calcium, Serum phosphorus, HbA1c, triglycerides, total cholesterol, CVD, hypertension, cancer, and medication usage. * P < 0.05, ** P < 0.01, *** P < 0.001; P < 0.05 was considered statistically significant.

Table S9 Association between ALI and femur BMD in T2DM (The medication usage was adjusted)

| Variable | Crude Model | Model 1 | Model 2 | Model3 |
| --- | --- | --- | --- | --- |
|  | β (95% CI) | β (95% CI) | β (95% CI) | β (95% CI) |
| Total femur BMD (g/cm^2^) |  |  |  |  |
| Log2-ALI | 0.03(0.01,0.04) **^**^** | 0.03(0.02,0.05) **^***^** | 0.03(0.01,0.05) **^***^** | 0.03(0.01,0.05) **^**^** |
| Q1 | -0.04(-0.08, 0.00) **^*^** | -0.06(-0.09, -0.03) **^***^** | -0.05(-0.09, -0.02) **^**^** | -0.05(-0.08, -0.02) **^**^** |
| Q2 | -0.03(-0.06, 0.00) **^*^** | -0.03(-0.04, 0.00) **^*^** | -0.02(-0.05, 0.00) | -0.03(-0.05, 0.00) **^*^** |
| Q3 | -0.02(-0.06, 0.02) | -0.03(-0.06, 0.01) | -0.03(-0.06, 0.00) | -0.03(-0.06, 0.00) |
| Q4 | Reference | Reference | Reference | Reference |
| P for trend | 0.04 | <0.01 | <0.01 | <0.01 |
| Femur neck BMD (g/cm^2^) |  |  |  |  |
| Log2-ALI | 0.02(0.01,0.03) **^***^** | 0.02(0.01,0.03) **^**^** | 0.02(0.01,0.03) **^**^** | 0.02(0.01,0.03) **^**^** |
| Q1 | -0.04(-0.07, -0.02) **^**^** | -0.04(-0.07, -0.02) **^**^** | -0.04(-0.06, -0.01) **^**^** | -0.04(-0.06, -0.01) **^**^** |
| Q2 | -0.05(-0.07, -0.02) **^***^** | -0.03(-0.05, -0.01) **^**^** | -0.02(-0.05, 0.00) **^*^** | -0.03(-0.05, 0.00) **^*^** |
| Q3 | -0.03(-0.07, 0.02) | -0.02(-0.07, 0.02) | -0.02(-0.05, 0.01) | -0.02(-0.05, 0.01) |
| Q4 | Reference | Reference | Reference | Reference |
| P for trend | <0.01 | <0.01 | <0.01 | <0.01 |
| Trochanter BMD (g/cm^2^) |  |  |  |  |
| Log2-ALI | 0.02(0.01,0.04) **^**^** | 0.03(0.01,0.04) **^***^** | 0.02(0.01,0.04) **^**^** | 0.02(0.01,0.04) **^**^** |
| Q1 | -0.03(-0.06, 0.00) | -0.05(-0.08, -0.02) **^**^** | -0.04(-0.07, -0.01) **^**^** | -0.04(-0.07, -0.01) **^*^** |
| Q2 | -0.02(-0.04, 0.01) | -0.02(-0.04, 0.01) | -0.01(-0.04, 0.01) | -0.01(-0.04, 0.01) |
| Q3 | 0.00(-0.03, 0.03) | -0.01(-0.04, 0.02) | -0.01(-0.04, 0.01) | -0.01(-0.04, 0.01) |
| Q4 | Reference | Reference | Reference | Reference |
| P for trend | 0.06 | <0.01 | <0.01 | 0.02 |
| Intertrochanter BMD (g/cm^2^) |  |  |  |  |
| Log2-ALI | 0.03(0.01,0.05) **^*^** | 0.03(0.01,0.05) **^**^** | 0.03(0.01,0.05) **^**^** | 0.03(0.01,0.05) **^**^** |
| Q1 | -0.04(-0.09, 0.01) | -0.06(-0.10, -0.03) **^**^** | -0.06(-0.10, -0.02) **^**^** | -0.06(-0.10, -0.02) **^**^** |
| Q2 | -0.04(-0.07, 0.00) | -0.03(-0.07, 0.00) **^*^** | -0.03(-0.06, 0.00) | -0.03(-0.06, 0.00) |
| Q3 | -0.03(-0.08, 0.02) | -0.04(-0.09, 0.00) | -0.04(-0.08, 0.00) | -0.04(-0.08, 0.00) |
| Q4 | Reference | Reference | Reference | Reference |
| P for trend | 0.10 | <0.01 | 0.01 | 0.02 |

95% CI, 95% confidence interval; OR, odds ratio; ALI, advanced lung cancer inflammation index. Crude Model: Not adjusted; Model 1 adjusted for age, sex, and race; Model 2 further adjusted for smoking status, marital status, education level, PIR, physical activity, and medication usage on the basis of Model 1; Model 3 additionally adjusted for AST, ALT, ALP, Serum creatinine, Serum uric acid, blood urea nitrogen, Serum calcium, Serum phosphorus, HbA1c, triglycerides, total cholesterol, CVD, hypertension, cancer, and medication usage. * P < 0.05, ** P < 0.01, *** P < 0.001; P < 0.05 was considered statistically significant.

Table S10 Baseline Characteristics of Participants with T2DM (Included CRP)

| **Characteristic** | **Total**,  N^1^ = 878 (100%)^2^ | **T2DM and OP**,  N^1^ = 387 (44%)^2^ | **T2DM and non-OP**,  N^1^ = 491 (56%)^2^ | **P Value**^3^ |
| --- | --- | --- | --- | --- |
| **Age (years)** | 64 (9) | 66 (9) | 62 (9) | **<0.001** |
| **Sex** |  |  |  | **<0.001** |
| *female* | 355 (42%) | 198 (54%) | 157 (33%) |  |
| *male* | 523 (58%) | 189 (46%) | 334 (67%) |  |
| **Race** |  |  |  | **0.005** |
| *Non-Hispanic White* | 396 (71.4%) | 193 (73%) | 203 (70%) |  |
| *Non-Hispanic Black* | 184 (11.4%) | 52 (6.6%) | 132 (15%) |  |
| *Mexican American* | 180 (7.1%) | 85 (7.4%) | 95 (7.0%) |  |
| *Other Race* | 118 (10.1%) | 57 (13%) | 61 (8.0%) |  |
| **PIR** |  |  |  | **0.037** |
| *High (>3.5)* | 264 (45%) | 97 (39%) | 167 (49%) |  |
| *Low (≤1.3)* | 242 (16%) | 118 (19%) | 124 (14%) |  |
| *Medium (>1.3, ≤3.5)* | 372 (39%) | 172 (41%) | 200 (37%) |  |
| **Smoking status** |  |  |  | 0.5 |
| *Current smoker* | 126 (12.4%) | 59 (13%) | 67 (12%) |  |
| *Former smoker* | 347 (38.4%) | 154 (40%) | 193 (37%) |  |
| *Never smoker* | 405 (49.2%) | 174 (47%) | 231 (51%) |  |
| **Education attainment** |  |  |  | 0.14 |
| *Less Than 9th Grade* | 173 (11%) | 92 (13.2%) | 81 (8.4%) |  |
| *9-11th Grade* | 133 (11%) | 50 (10.4%) | 83 (11.3%) |  |
| *High School Grad/GED* | 218 (28%) | 96 (27.9%) | 122 (28.2%) |  |
| *Some College or AA degree* | 206 (27%) | 97 (29.4%) | 109 (25.4%) |  |
| *College Graduate or above* | 148 (23%) | 52 (19.1%) | 96 (26.7%) |  |
| **Marital status** |  |  |  | **<0.001** |
| *Married/cohabiting* | 563 (69%) | 219 (62%) | 344 (74%) |  |
| *Never married* | 49 (5.0%) | 14 (3.0%) | 35 (6.0%) |  |
| *Widowed/divorced/separated* | 266 (26%) | 154 (35%) | 112 (20%) |  |
| **Physical activity** |  |  |  | 0.6 |
| *Active* | 251 (31%) | 120 (33%) | 131 (30%) |  |
| *Inactive* | 627 (69%) | 267 (67%) | 360 (70%) |  |
| **CVD** | 217 (24%) | 99 (22.9%) | 118 (24.3%) | 0.7 |
| **Hypertension** | 646 (72%) | 290 (73.1%) | 356 (71.5%) | 0.6 |
| **Cancer** | 147 (19%) | 67 (20.5%) | 80 (17.4%) | 0.4 |
| **HbA1c (%)** | 6.86 (1.38) | 6.86 (1.38) | 6.86 (1.38) | >0.9 |
| **BMI (kg/m^2^)** | 30.5 (5.6) | 28.6 (5.3) | 32.1 (5.3) | **<0.001** |
| **CRP (mg/dL)** | 0.43 (0.68) | 0.35 (0.48) | 0.48 (0.81) | **0.005** |
| **ALT (U/L)** | 28 (30) | 26 (15) | 29 (38) | 0.11 |
| **AST (U/L)** | 27 (16) | 26 (11) | 27 (20) | 0.4 |
| **ALP (U/L)** | 72 (23) | 73 (22) | 72 (23) | 0.6 |
| **Blood urea nitrogen (mg/dL)** | 15.4 (6.0) | 15.9 (6.6) | 15.0 (5.3) | 0.072 |
| **Serum Uric acid(mg/dL)** | 5.77 (1.44) | 5.47 (1.38) | 6.01 (1.44) | **<0.001** |
| **Serum creatinine (µmol/L)** | 0.98 (0.47) | 0.97 (0.57) | 0.98 (0.38) | 0.8 |
| **Serum Calcium (mg/dL)** | 9.46 (0.33) | 9.47 (0.34) | 9.46 (0.32) | 0.7 |
| **Serum Phosphorus (mg/dL)** | 3.72 (0.56) | 3.76 (0.61) | 3.69 (0.52) | 0.10 |
| **Total Cholesterol** **(mg/dL)** | 191 (45) | 195 (47) | 188 (43) | 0.14 |
| **Triglyceride (mg/dL)** | 192 (151) | 194 (159) | 190 (145) | 0.9 |
| **Log2-ALI** | 5.93 (0.74) | 5.76 (0.79) | 6.06 (0.67) | **<0.001** |
| **Total femur** | 0.97 (0.17) | 0.84 (0.11) | 1.08 (0.13) | **<0.001** |
| **Femur neck** | 0.81 (0.15) | 0.68 (0.07) | 0.90 (0.11) | **<0.001** |
| **Trochanter** | 0.74 (0.15) | 0.63 (0.10) | 0.83 (0.13) | **<0.001** |
| **Intertrochanter** | 1.15 (0.20) | 1.00 (0.14) | 1.27 (0.15) | **<0.001** |
| ^1^N not Missing (unweighted);^2^Mean ± SD for continuous; n (%) for categorical;^3^t-test adapted to complex survey samples; chi-squared test with Rao & Scott's second-order correction. PIR, poverty income ratio; CRP, C-reactive protein; CVD, cardiovascular disease; HbA1c, glycated hemoglobin; ALT, alanine aminotransferase; AST, aspartate aminotransferase; ALP, alkaline phosphatase; ALI, advanced lung cancer inflammation index; OP, osteoporosis; T2DM, type 2 Diabetes mellitus. Bold values indicate p-value < 0.05. | | | | |
|  | | | | |
|  | | | | |

Table S11 Association between ALI and CRP in T2DM

| Variable | Crude Model | Model 1 | Model 2 | Model3 |
| --- | --- | --- | --- | --- |
|  | OR (95% CI) | OR (95% CI) | OR (95% CI) | OR (95% CI) |
| CRP | -0.10(-0.19, -0.02) * | -0.14(-0.23, -0.05) ** | -0.14(-0.22, -0.05) ** | -0.13(-0.22, -0.05) ** |
| Q1 | Reference | Reference | Reference | Reference |
| Q2 | -0.15(-0.29, -0.01) * | -0.15(-0.30, -0.01) * | -0.15(-0.29, 0.00) * | -0.14(-0.28, 0.01) |
| Q3 | -0.23(-0.38, -0.09) ** | -0.26(-0.40, -0.11) ** | -0.25(-0.40, -0.10) ** | -0.24(-0.36, -0.11) ** |
| Q4 | -0.18(-0.36, 0.01) | -0.25(-0.42, -0.07) ** | -0.24(-0.42, -0.07) ** | -0.25(-0.40, -0.09) ** |
| P for trend | 0.04 | <0.01 | <0.01 | <0.01 |

95% CI, 95% confidence interval; OR, odds ratio; CRP, C-reactive protein. Crude Model: Not adjusted; Model 1 adjusted for age, sex, and race; Model 2 further adjusted for smoking status, marital status, education level, PIR and physical activity on the basis of Model 1; Model 3 additionally adjusted for AST, ALT, ALP, Serum creatinine, Serum uric acid, blood urea nitrogen, Serum calcium, Serum phosphorus, HbA1c, triglycerides, total cholesterol, CVD, hypertension, and cancer on the basis of Model 2. * P < 0.05, ** P < 0.01, *** P < 0.001; P < 0.05 was considered statistically significant.


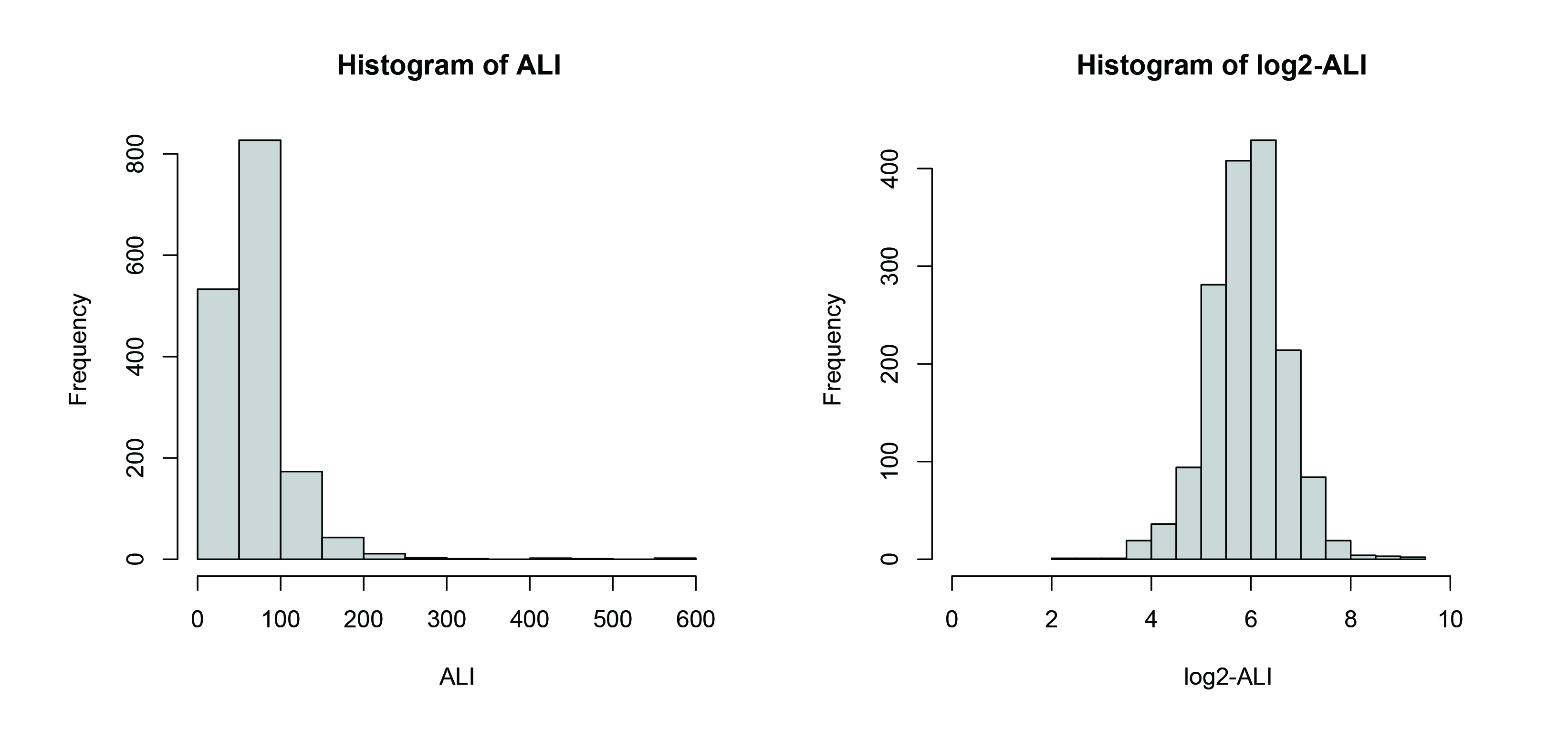


Figure S1 The distribution of the ALI and the distribution of the log2-transformed ALI. ALI, advanced lung cancer inflammation index.
